# GENDER DIFFERENCES IN MUSCLE STRENGTH AND TISSUE ASYMMETRY CHANGES OVER FIVE YEARS AMONG OLDER ADULTS

**DOI:** 10.1093/geroni/igae098.2821

**Published:** 2024-12-31

**Authors:** Milan Chang (Gudjonsson), Marco Recenti, Carlo Ricciardi, Alfonso Maria Ponsiglione, Francesco Amato, Magnus Kjartan Gislason, Paolo Gargiulo

**Affiliations:** Landspitali University Hospital, Seltjarnarnes, Hofuoborgarsvaoio, Iceland; Reykjavik University, Reykjavik, Hofuoborgarsvaoio, Iceland; University of Naples Federico II, Naples, Calabria, Italy; University of Naples Federico II, Naples, Campania, Italy; Università degli Studi Magna Græcia di Catanzaro, Napoli, Campania, Italy; Reykjavik University, Reykjavik, Hofuoborgarsvaoio, Iceland; Reykjavik University, Reykjavik, Hofuoborgarsvaoio, Iceland

## Abstract

**Background:**

The declines in muscle strength during aging process is linked to reductions in tissue integrity and underlying cellular processes. It suggests potential gender differences in these fundamental mechanisms. The current study investigates whether the decline in muscle strength over a 5-year period correlates with changes in tissue asymmetry and explores potential gender differences between men and women.

**Methods:**

The longitudinal study comprised 3156 participants residing in Reykjavik, Iceland, with a baseline mean age of 74.9. The analysis included 1,150 men and 1,587 women (n=2,737). Tissue asymmetry, assessed through CT scans, was defined by differences between the right and left sides across 11 soft tissue composition parameters for fat, muscle, and connective tissue. Changes in leg strength and asymmetry parameters over 5 years were calculated by subtracting follow-up values from baseline. Linear regression analysis was performed to assess the association between changes of maximum knee extension strengths and changes in soft-tissue asymmetry over 5 years, adjusting for confounders by gender.

**Results:**

In men, a decline in leg strength over 5 years was significantly associated with an increased asymmetry across five parameters (2 related to fat and 3 related to muscle (p< 0.05)). In women, associations were observed with an increased asymmetry parameters involving fat, muscle, and connective tissue (p< 0.03).

**Conclusion:**

Gender-specific variations in asymmetry parameters highlight how fat and muscle composition in men uniquely contribute to leg strength changes over time. This emphasizes the need to consider gender-specific factors in understanding age-related muscle strength changes, informing targeted interventions for older adults.

